# Body Fat Distribution and the Risk of Incident Metabolic Syndrome: A Longitudinal Cohort Study

**DOI:** 10.1038/s41598-017-09723-y

**Published:** 2017-09-08

**Authors:** Hyuktae Kwon, Donghee Kim, Joo Sung Kim

**Affiliations:** 1 Department of Family Medicine, Healthcare Research Institute, Seoul National University Hospital Healthcare System Gangnam Center, Seoul, Korea; 2 Department of Family Medicine, Seoul National University Hospital & College of Medicine, Seoul, Korea; 3 Department of Internal Medicine, Healthcare Research Institute, Seoul National University Hospital Healthcare System Gangnam Center, Seoul, Korea; 4 Division of Gastroenterology and Hepatology, Stanford University School of Medicine, Stanford, California, United States

## Abstract

The effect of visceral adipose tissue (VAT) and subcutaneous adipose tissue (SAT) area on metabolic syndrome (MS) has been debated. We aimed to evaluate the effects of VAT and SAT on the incidence of MS and its components in a large and apparently healthy Asian population. We performed a longitudinal cohort study of 1,964 subjects who received health screenings over a 5-year follow-up period; 317 incidents of MS (16.1%) were observed during a median follow-up of 4.5 years. The VAT area was significantly associated with a higher incidence of MS; the adjusted HR for incident MS per 1 SD of VAT was 1.50 (95% CI 1.29–1.74), and the adjusted HR of the 5^th^ VAT quintile compared with the 1^st^ quintile was 3.73 (95% CI 2.22–6.28). However, the SAT area was not associated with incident MS. Although the VAT area was longitudinally associated with the incidence of each component of MS, the SAT area was inversely associated with the risk of high blood pressure, fasting blood sugar, and triglycerides, with marginal significance. In conclusion, the VAT area is longitudinally associated with an increased risk of incident MS, while SAT may have a protective effect against the incidence of individual MS components.

## Introduction

According to a report from the World Health Organization (WHO), the worldwide prevalence of obesity nearly doubled between 1980 and 2014; in 2014, 38% of men and 40% of women were overweight and 11% of men and 15% of women were obese^1^. Obesity, especially abdominal obesity, is associated with insulin resistance, which in turn leads to hyperglycemia, hypertension, dyslipidemia, and other metabolic abnormalities^2^. Metabolic syndrome (MS) is characterized by clusters of metabolic abnormalities and has been associated with the incidence of type 2 diabetes as well as cardiovascular morbidity and mortality^3, 4^. The increasing prevalence of obesity and type 2 diabetes has led to an increased prevalence of MS, which has a substantial impact on public health^5, 6^. Therefore, identifying subjects who have a high risk of MS is important in clinical practice. Early intervention via lifestyle modification may prevent incident MS in subjects without MS and thereby reduce clinical burden.

Based on previous studies, regional body fat distribution, regardless of general obesity, may play a critical role in MS^7^. Waist circumference (WC), which is a component of MS, does not sufficiently discriminate between visceral adipose tissue (VAT) and subcutaneous adipose tissue (SAT) and has shown stronger associations with SAT than with VAT^8^. Both VAT and SAT contribute to abdominal obesity; however, there has been debate regarding their effect on MS. Several cross-sectional studies have reported a relatively consistent relationship between VAT, MS, and insulin resistance^9–12^, but the association between SAT and MS remains controversial^9–11, 13–16^. While VAT involves an active endocrine organ that releases numerous adipokines and hormones that regulate metabolism and inflammation, SAT may be protective against the development of metabolic abnormalities as a ‘metabolic sink’^17, 18^.

Previous studies, including ours, on the associations between body fat distribution and MS have mainly been cross-sectional^9–13, 19, 20^; therefore, the results do not enable the determination of cause and effect relationships. Several longitudinal studies on these associations have recently been published^16, 21–23^ but were subject to other limitations. One study from Japan assessed employees who lacked MS components at baseline at only one company and did not consider several important variables, such as alcohol intake and physical activity^16^. The Framingham Offspring Study comprised mostly Caucasians, and the results therefore cannot be generalized to other ethnicities^21, 22^. In addition, the MESA cohort study did not test the association of individual components of MS with VAT^23^.

The purpose of the present study was to evaluate the effects of VAT and SAT on the incidence of MS and its components by adjusting for traditional metabolic risk factors in a large and apparently healthy Asian population.

## Results

### General characteristics of the study population

As outlined in the Methods section, of the 5,100 subjects in the baseline cohort, 2,581 (50.6%) received voluntarily health screening check-ups during the approximately 5-year follow-up. To test the selection process, subjects with follow-up data were compared to those who were lost to follow-up (Supplementary Table 1). No difference was observed between the groups in anthropometric or laboratory variables except for gender, diabetes, and the SAT area. Considering the similar total abdominal fat values in the two groups, the differences in the VAT and SAT areas between the two groups were mainly due to differences in gender distribution.

The clinical characteristics of the baseline cohort according to the incidence of MS are presented in Table 1. Of the subjects without MS at baseline, 16.1% (317/1964) developed MS during the median 4.5 years of follow-up. Comparisons of baseline characteristics revealed that most of the clinical variables, including gender, BMI, WC, change in WC, TG, HDL cholesterol, fasting glucose, and HOMA-IR, were less metabolically favorable in subjects with incident MS than in those without MS. Subjects with incident MS were more likely to be older, hypertensive, and diabetic and to currently smoke. Subjects who developed MS had greater VAT (142.3 cm^2^ vs. 100.2 cm^2^) and SAT areas (150.9 cm^2^ vs. 137.2 cm^2^).

**Table 1:** Baseline characteristics of subjects according to the development of MS (n = 1,964).

	No development of MS (n = 1,647)	Development of MS (n = 317)	*P-*value
Age (years)	50.0 ± 8.6	52.1 ± 8.9	<0.001
Male (%)	1007 (61.1)	260 (82.0)	<0.001
Smoking (%)	517 (31.4)	132 (41.6)	0.001
Diabetes mellitus (%)	46 (2.8)	16 (5.0)	0.036
Diabetes medication (%)	32 (1.9)	12 (3.8)	0.042
Hypertension (%)	176 (10.7)	83 (26.2)	<0.001
Systolic BP (mmHg)	113.6 ± 13.2	119.6 ± 12.5	<0.001
Diastolic BP (mmHg)	73.4 ± 0.7	78.1 ± 9.5	<0.001
Hypertension medication (%)	138 (8.4)	76 (24.0)	<0.001
Lipid-lowering medication (%)	47 (2.9)	48 (15.1)	<0.001
BMI (kg/m^2^)	22.96 ± 2.51	24.91 ± 2.37	<0.001
WC (cm)	82.93 ± 7.04	88.25 ± 6.10	<0.001
Change in WC (cm)	−0.15 ± 3.80	1.35 ± 3.72	<0.001
Cholesterol (mg/dL)	190.4 ± 31.1	202.0 ± 35.7	<0.001
TG (mg/dL)	95.7 ± 46.4	125.1 ± 52.6	<0.001
HDL cholesterol (mg/dL)	56.6 ± 13.7	51.2 ± 12.7	<0.001
Fasting glucose (mg/dL)	91.6 ± 12.7	97.0 ± 15.4	<0.001
HOMA-IR	1.82 ± 0.82	2.23 ± 1.02	<0.001
VAT (cm^2^)	100.2 ± 47.6	142.3 ± 46.9	<0.001
SAT (cm^2^)	137.2 ± 52.4	150.9 ± 50.4	<0.001

The data are shown as the mean ± SD or n (%).

### Adipose tissue area and incident MS

As shown in Table 2, increasing VAT areas were associated with an increasing incidence of MS in a dose-dependent manner. In the age- and sex-adjusted models, the HRs for incident MS in subjects in quintiles 3, 4, and 5 compared to subjects in quintile 1 of VAT area were 2.86 (95% CI 1.85–4.43), 4.27 (95% CI 2.78–6.54), and 5.50 (95% CI 3.59–8.43), respectively. In addition, BMI, smoking, alcohol consumption, menopausal status, hormone replacement therapy (HRT), soft drink consumption, physical activity, and the SAT area did not lead to reduced HRs. The adjusted HRs for the VAT area by quintiles were 3.10 (95% CI 1.92–4.98) for the 4^th^ quintile and 3.37 (95% CI 2.01–5.66) for the 5^th^ quintile compared with the 1^st^ quintile (*p* for trend <0.001). Adding the change in WC (model 2) and changes in other covariates to the model (model 3) did not significantly attenuate the HRs. The adjusted HRs of subjects in the 4^th^ and 5^th^ quintiles of the VAT area compared to the subjects in the lowest quintile were 3.34 (95% CI 2.07–5.39) and 3.73 (95% CI 2.22–6.28), respectively (*p* for trend <0.001). The results were similar when adjusting for changes in BMI and body weight (data not shown). This association persisted after adjusting for systolic BP, fasting glucose, TG, and HDL cholesterol levels (Supplementary Table 2). Because insulin resistance plays an important role in the development of MS, the HOMA-IR was included in the analysis. A similar association was observed after adjusting for the HOMA-IR (Supplementary Table 2). The effect of the interaction between gender and VAT or SAT on incident MS was also assessed. No significant interactions between gender and VAT or SAT regarding incident MS were found.

**Table 2:** Multivariable analyses of the risk of incident MS in subjects without MS at baseline (n = 1964).

	Multivariable model 1		Multivariable model 2		Multivariable model 3	
	HR (95% CI)	*P-*value	HR (95% CI)	*P-*value	HR (95% CI)	*P-*value
VAT Quintiles						
Q1	1	<0.001*	1	<0.001*	1	<0.001*
Q2	1.75 (1.09–2.79)	0.021	1.88 (1.17–3.02)	0.009	1.98 (1.23–3.18)	0.005
Q3	2.29 (1.43–3.67)	0.001	2.53 (1.58–4.04)	<0.001	2.57 (1.60–4.13)	<0.001
Q4	3.10 (1.92–4.98)	<0.001	3.31 (2.05–5.33)	<0.001	3.34 (2.07–5.39)	<0.001
Q5	3.37 (2.01–5.66)	<0.001	3.78 (2.26–6.35)	<0.001	3.73 (2.22–6.28)	<0.001
VAT (per 1 SD)	1.51 (1.30–1.76)	<0.001	1.51 (1.31–1.75)	<0.001	1.50 (1.29–1.74)	<0.001
SAT Quintiles						
Q1	1	0.757*	1	0.717*	1	0.580*
Q2	0.87 (0.57–1.33)	0.524	0.83 (0.54–1.27)	0.386	0.80 (0.53–1.23)	0.312
Q3	1.03 (0.68–1.56)	0.878	0.99 (0.65–1.50)	0.963	1.00 (0.66–1.52)	0.995
Q4	0.95 (0.61–1.47)	0.814	0.91 (0.58–1.41)	0.660	0.89 (0.57–1.39)	0.622
Q5	1.03 (0.62–1.70)	0.906	1.03 (0.63–1.70)	0.901	1.08 (0.65–1.79)	0.769
SAT (per 1 SD)	0.94 (0.79–1.11)	0.468	0.96 (0.81–1.14)	0.648	0.98 (0.82–1.17)	0.849
Age	1.02 (1.00–1.03)	0.046	1.02 (1.00–1.03)	0.029	1.02 (1.01–1.04)	0.011
Sex	2.17 (1.38–3.41)	0.001	2.12 (1.35–3.34)	0.001	2.29 (1.39–3.78)	0.001
BMI	1.11 (1.04–1.20)	0.003	1.11 (1.04–1.19)	0.004	1.11 (1.03–1.19)	0.005
Smoking	0.83 (0.65–1.06)	0.130	0.86 (0.67–1.10)	0.239	0.82 (0.63–1.07)	0.141
Alcohol	1.23 (0.94–1.60)	0.129	1.28 (0.98–1.67)	0.070	1.40 (1.05–1.86)	0.020
Menopause	1.36 (0.77–2.39)	0.288	1.63 (0.92–2.88)	0.092	1.81 (1.00–3.28)	0.051
HRT	0.34 (0.10–1.11)	0.074	0.29 (0.09–0.95)	0.041	0.27 (0.08–0.88)	0.030
Soft drink consumption	0.81 (0.57–1.16)	0.257	0.84 (0.58–1.19)	0.323	0.85 (0.59–1.23)	0.393
Physical Activity						
Inactive (0 MET/wk)	1		1		1	
>0–500 MET/wk	1.01 (0.71–1.44)	0.946	0.92 (0.64–1.31)	0.625	0.87 (0.61–1.25)	0.459
500–1000 MET/wk	0.84 (0.61–1.14)	0.255	0.74 (0.54–1.02)	0.065	0.74 (0.54–1.02)	0.064
>1000 MET/wk	0.85 (0.63–1.14)	0.280	0.75 (0.56–1.02)	0.064	0.75 (0.55–1.01)	0.057
Change in WC (per 1 cm)			1.11 (1.07–1.14)	<0.001	1.11 (1.08–1.14)	<0.001
New smoking					0.77 (0.49–1.22)	0.772
New menopause					4.16 (1.27–13.61)	0.018
New HRT					0.61 (0.13–2.90)	0.531
New alcohol intake					1.46 (1.04–2.05)	0.030
New soft drink consumption					0.91 (0.64–1.29)	0.590

*P-value for test of trend in odds. Multivariable model 1 was adjusted for age, sex, BMI, smoking status, excessive alcohol consumption, menopausal status, HRT, soft drink consumption, physical activity, VAT area, and SAT area. Multivariable model 2 included the change in WC in addition to the variables addressed in model 1. Multivariable model 3 included new smoking, new menopause, new HRT, new alcohol intake, and new soft drink consumption during follow-up in addition to the variables addressed in model 2. VAT area (cm^2^): men: Q1, 12.51~96.36; Q2, ~124.77; Q3, ~151.20; Q4, ~180.75; Q5, ~318.11; women: Q1, 10.40~43.42; Q2, ~63.93; Q3, ~87.06; Q4, 113.59; Q5, ~238.41. SAT area (cm^2^): men: Q1, 9.66~97.62; Q2, ~119.88; Q3, ~141.42; Q4, ~174.40; Q5, ~461.98; women: Q1, 28.29~117.45; Q2, ~149.98; Q3, ~179.48; Q4, ~220.68; Q5, ~397.31.

The SAT area was associated with incident MS in the age- and sex-adjusted model. However, this association was non-significant when adjusting for other risk factors.

Subjects with incident MS had a greater increase in WC during the follow-up period. After adjusting for multiple variables, a 1-cm increase in WC during follow-up was associated with an 11% increase in incident MS (HR 1.11, 95% CI 1.08–1.14).

We conducted several sensitivity analyses to examine the robustness of our findings (Table 3). Analyses were restricted to those who did not have any components of MS at baseline. Of the subjects without individual components of MS at baseline, 11.3% (67/588) developed MS during the follow-up. In the age- and sex-adjusted model, the HR for incident MS per 1-SD in VAT area was 2.24 (95% CI 1.64–3.6, *p* < 0.001). After adjustments for multiple risk factors, including SAT, the HR for incident MS was slightly attenuated but remained significant (HR per 1-SD, 2.04 [95% CI 1.48–2.81]). This association persisted after adjusting for changes in WC during the follow-up.

**Table 3:** Multivariable analyses of the risk of incident MS in subjects without any component of MS at baseline (n = 588).

	Age, sex adjusted model		Multivariable model 1		Multivariable model 2	
	HR (95% CI)	*P-*value	HR (95% CI)	*P-*value	HR (95% CI)	*P-*value
VAT (per 1 SD)	2.24 (1.64–3.06)	<0.001	2.04 (1.48–2.81)	<0.001	2.08 (1.51–2.85)	<0.001
SAT(per 1 SD)	1.03 (0.71–1.49)	0.893	0.75 (0.49–1.15)	0.181	0.69 (0.45–1.06)	0.090
Age			1.00 (0.96–1.04)	0.887	1.01 (0.97–1.05)	0.793
Sex			0.75 (0.30–1.88)	0.541	0.70 (0.28–1.72)	0.435
BMI			1.31 (1.07–1.60)	0.009	1.31 (1.07–1.60)	0.008
Menopause			3.18 (1.34–7.55)	0.006	2.95 (1.22–7.09)	0.016
WC change					1.05 (1.02–1.07)	<0.001

Multivariable model 1 was adjusted for age, sex, BMI, menopause, VAT area, and SAT area. Multivariable model 2 included the change in WC in addition to the variables addressed in model 1.

### Risk factors of incident MS

Age (HR 1.02, 95% CI 1.01–1.04), male sex (HR 2.29, 95% CI 1.39–3.78), alcohol consumption (HR 1.40, 95% CI 1.05–1.86) and HRT (HR 0.27, 95% CI 0.08–0.88) were significantly associated with incident MS (Table 2). Although female sex and HRT showed protective effects, smoking was not associated with incident MS. Menopause (HR 1.81, 95% CI 1.00–3.28) was marginally associated with incident MS. Increasing physical activity was inversely associated with incident MS compared with physical inactivity.

### Association between adipose tissue areas and the incidence of individual components of MS

As shown in Table 4, the HRs for the incidence of the dichotomous components of MS for each 1 SD increase in VAT area were 1.24 (95% CI 1.04–1.48, *p* for trend = 0.047) for high BP, 1.26 (95% CI 1.07–1.47, *p* for trend = 0.003) for high fasting glucose (≥100 mg/dL or taking antidiabetic medication), 1.22 (95% CI 1.07–1.38, *p* for trend <0.001) for low HDL cholesterol, and 1.55 (95% CI 1.31–1.83, *p* for trend <0.001) for high TG. Regarding the SAT area, the HR for the incidence of the dichotomous components of MS for each 1-SD increase in SAT area was 1.24 (95% CI 1.08–1.43, *p* for trend = 0.001) for low HDL cholesterol. However, the SAT area was protective against the incidence of high BP (*p* for trend = 0.05), high fasting glucose (HR for 1-SD increment 0.85 95% CI 0.72–1.00) and high TG (HR for 1-SD increment of 0.90 95% CI 0.76–1.06), with marginal significance in multivariate model 1 and after adjustment for change in WC (model 2). However, after further adjustment for change in lifestyle, these associations were attenuated. These results suggest that the longitudinal protective effect of SAT on some individual components of MS might be partially attenuated by worsening lifestyles such as onset of smoking or alcohol or soft drink consumption.

**Table 4:** Incidence of each component of MS by the VAT and SAT areas at baseline.

	VAT					P for	VAT	SAT					P for	SAT
	Q1	Q2	Q3	Q4	Q5	Trend^*^	(per 1 SD)	Q1	Q2	Q3	Q4	Q5	Trend^*^	(per 1 SD)
**High BP**														
Model 1	1	0.89 (0.58–1.37)	1.36 (0.90–2.04)	1.58 (1.02–2.45)	1.52 (0.94–2.44)	0.015	1.24 (1.06–1.45)	1	0.87 (0.58–1.31)	0.81 (0.53–1.24)	0.76 (0.49–1.18)	0.57 (0.34–0.98)	0.050	0.83 (0.69–1.01)
Model 2	1	0.91 (0.59–1.40)	1.40 (0.93–2.11)	1.58 (1.02–2.45)	1.55 (0.97–2.50)	0.014	1.24 (1.06–1.44)	1	0.85 (0.56–1.28)	0.81 (0.53–1.23)	0.75 (0.48–1.17)	0.56 (0.33–0.96)	0.048	0.84 (0.70–1.02)
Model 3	1	0.87 (0.56–1.35)	1.42 (0.92–2.19)	1.58 (0.99–2.51)	1.39 (0.82–2.36)	0.047	1.24 (1.04–1.48)	1	0.83 (0.54–1.28)	0.78 (0.50–1.23)	0.75 (0.47–1.20)	0.64 (0.35–1.15)	0.149	0.86 (0.69–1.07)
**High FBS**														
Model 1	1	1.23 (0.84–1.81)	1.62 (1.10–2.39)	1.92 (1.28–2.87)	2.00 (1.28–3.13)	<0.001	1.30 (1.13–1.50)	1	0.89 (0.62–1.29)	0.92 (0.63–1.33)	0.73 (0.49–1.09)	0.76 (0.48–1.19)	0.148	0.85 (0.72–1.00)
Model 2	1	1.28 (0.88–1.88)	1.72 (1.17–2.54)	2.04 (1.36–3.06)	2.05 (1.31–3.22)	<0.001	1.30 (1.12–1.50)	1	0.87 (0.60–1.26)	0.90 (0.63–1.31)	0.72 (0.48–1.07)	0.73 (0.46–1.14)	0.113	0.85 (0.72–1.00)
Model 3	1	1.26 (0.85–1.86)	1.67 (1.11–2.51)	2.06 (1.34–3.15)	1.77 (1.08–2.90)	0.003	1.26 (1.07–1.47)	1	0.83 (0.57–1.22)	0.97 (0.66–1.42)	0.69 (0.45–1.07)	0.84 (0.51–1.37)	0.341	0.89 (0.74–1.06)
**Low HDL**														
Model 1	1	1.57 (1.12–2.18)	1.92 (1.37–2.68)	2.35 (1.68–3.30)	1.90 (1.30–2.76)	<0.001	1.17 (1.06–1.31)	1	1.28 (0.93–1.75)	1.36 (0.99–1.87)	1.48 (1.07–2.05)	1.57 (1.10–2.23)	0.015	1.13 (1.01–1.26)
Model 2	1	1.59 (1.14–2.22)	1.96 (1.40–2.74)	2.39 (1.70–3.35)	1.94 (1.33–2.82)	<0.001	1.17 (1.05–1.30)	1	1.27 (0.92–1.74)	1.36 (0.99–1.87)	1.47 (1.06–2.04)	1.56 (1.10–2.23)	0.015	1.13 (1.01–1.26)
Model 3	1	1.65 (1.16–2.34)	2.05 (1.43–2.94)	2.49 (1.73–3.59)	2.00 (1.30–3.07)	<0.001	1.22 (1.07–1.38)	1	1.28 (0.89–1.82)	1.57 (1.10–2.25)	1.67 (1.15–2.43)	1.90 (1.24–2.90)	0.001	1.24 (1.08–1.43)
**High TG**														
Model 1	1	1.60 (1.05–2.34)	1.61 (1.05–2.46)	2.81 (1.84–4.31)	2.52 (1.54–4.10)	<0.001	1.43 (1.23–1.66)	1	0.74 (0.50–1.10)	0.96 (0.65–1.41)	0.76 (0.50–1.16)	0.80 (0.49–1.28)	0.476	0.90 (0.76–1.06)
Model 2	1	1.60 (1.08–2.39)	1.66 (1.09–2.54)	2.84 (1.86–4.35)	2.63 (1.61–4.29)	<0.001	1.43 (1.23–1.67)	1	0.72 (0.49–1.07)	0.95 (0.65–1.40)	0.75 (0.49–1.14)	0.80 (0.50–1.29)	0.519	0.90 (0.76–1.07)
Model 3	1	1.81 (1.19–2.75)	1.90 (1.22–2.97)	3.35 (2.14–5.25)	3.09 (1.81–5.26)	<0.001	1.55 (1.31–1.83)	1	0.71 (0.47–1.07)	1.00 (0.66–1.51)	0.82 (0.52–1.28)	0.82 (0.52–1.28)	0.967	0.94 (0.77–1.13)

Multivariable model 1 was adjusted for age, sex, BMI, smoking status, excessive alcohol consumption, menopausal status, HRT, soft drink consumption, physical activity, VAT area, and SAT area. Multivariable model 2 included the change in WC in addition to the variables addressed in model 1. Multivariable model 3 included new smoking, new menopause, new HRT, new alcohol intake, and new soft drink consumption during follow-up in addition to the variables addressed in model 2. VAT area (cm^2^): men: Q1, 12.51~96.36; Q2, ~124.77; Q3, ~151.20; Q4, ~180.75; Q5, ~318.11; women: Q1, 10.40~43.42; Q2, ~63.93; Q3, ~87.06; Q4, 113.59; Q5, ~238.41. SAT area (cm^2^): men: Q1, 9.66~97.62; Q2, ~119.88; Q3, ~141.42; Q4, ~174.40; Q5, ~461.98; women: Q1, 28.29~117.45; Q2, ~149.98; Q3, ~179.48; Q4, ~220.68; Q5, ~397.31.

## Discussion

In this large prospective study, the VAT area was longitudinally associated with incident MS and its components during a 5-year follow-up period. This association remained significant after adjusting for possible metabolic risk factors. In addition, the baseline SAT area was higher in subjects who had a reduced risk of some individual components of MS (high BP, fasting glucose) than in subjects who did not have a reduced risk, regardless of their baseline VAT area. This finding suggests that SAT may be a possible ‘metabolic sink’ for metabolic abnormalities.

Many cross-sectional studies have demonstrated that VAT is a risk factor for MS in different ethnicities^12, 14, 16, 23, 24^. However, the results of studies on the effect of SAT on MS have been inconsistent. Carr *et al*. revealed that SAT is associated with MS (OR 2.12 per 1-SD of SAT), but they adjusted for only age and sex^25^. Research from the Framingham Heart Study revealed that SAT was associated with MS after further adjustment for BMI, but they did not consider the effect of VAT^12^. The Dallas Heart Study reported that SAT was not associated with MS after adjustment for VAT and BMI, while VAT was significantly associated with MS^9^. However, in our previous study, SAT was inversely correlated with MS after adjusting for the SAT/VAT ratio. In this study, we found that the baseline SAT area was inversely associated with an increased risk of the individual components of MS^19^. This association may provide a possible link regarding the protective effect of SAT on MS.

However, in many longitudinal studies, such as the MERLOT study, SAT was not associated with incident MS after adjustment for BMI, while VAT was significantly associated with incident MS even after adjustment for SAT^16^. Similarly, in the Framingham Offspring Study, only VAT, not SAT, was associated with incident MS after adjustment for BMI and multiple risk factors^21^. In accordance with these prospective studies, our study revealed that only VAT, not SAT, increased the risk of incident MS when BMI, baseline fat tissue, and other traditional risk factors were considered. However, regarding individual components of MS, both VAT and SAT were positively associated with new onset hypertension only and showed no association with the incidence of diabetes, low HDL, or hypertriglyceridemia in the Framingham Offspring Study^21^. Our study revealed that VAT was positively associated with the incidence of each component of MS, while SAT was inversely associated with the incidence of high BP, high fasting glucose, and high TG, with marginal significance. While the criteria for hypertension and diabetes used in the Framingham Offspring Study were slightly different from our MS criteria for high BP and high fasting glucose, this difference in findings could be due to ethnic differences, and the results could indicate that SAT may be a ‘metabolic sink’ for metabolic abnormalities in Asians.

VAT is known to play a significant role in MS through various pathways^26^. Ectopic VAT accumulation can cause dysfunctional alterations in adipose tissue, such as free fatty acid metabolism changes^17^ and cellular hypoxia^27^. Another possible mechanism is through adipokines. Visceral obesity results in hypoadiponectinemia and an increase in tumor necrosis factor-α, interleukin (IL)-6, and other adipokines, which in turn result in insulin resistance^28^. Additionally, it is well known that VAT secretes more proinflammatory molecules, such as complement C3 and tumor necrosis factor-α, than SAT^29^. VAT also induces increased lipolysis and free fatty acids, which also cause insulin resistance^25, 30^.

Both VAT and SAT secrete various proinflammatory molecules that could result in insulin resistance. Recent research has indicated that VAT is an ‘ectopic fat’ that originates from the ‘overflow’ of fat beyond the capacity of SAT to store extra energy. In this theory, when SAT reaches its limit to store extra energy, these excess TG molecules will accumulate at undesired sites, such as VAT^26^. Therefore, peripheral SAT may exert a protective effect by decreasing fat deposition in the liver, muscle, heart, and VAT^26^. The therapeutic effect of thiazolidinedione is also explained by the redistribution of fat from pathogenic VAT to less-pathogenic SAT^31^. Recently, differential effects of deep and superficial SAT on metabolic risk factors have also been reported^32–34^. The marginal effect of SAT on metabolic abnormalities in our study may be explained by the different effects of deep SAT and superficial SAT. Further studies are needed to better understand these relationships.

The strengths of this longitudinal study are the use of CT-measured abdominal adiposity, the high-quality anthropometric data that followed a systemic protocol, the numerous metabolic variables included, and the large population size. Moreover, the study subjects may be representative of the general population given the nature of the health check-ups.

There are some limitations of this study. First, 50% of the subjects at baseline were not available at the 5-year follow-up. Although the subjects at baseline included more men and more often had diabetes than the subjects at follow-up, they nonetheless had the same values for BMI, WC, and lipid profiles. Therefore, the risk of selection bias in our study was mitigated. Second, we could not analyze the change in adipose tissue area at follow-up; however, we included change in WC in the model to minimize the effect of the change in adipose tissue area on incident MS. Third, we did not differentiate deep SAT from superficial SAT, which could have resulted in different associations of SAT and MS components.

In summary, our longitudinal cohort study revealed that an increased VAT area is longitudinally associated with an increased risk of incident MS, while SAT may have possible protective effects on the incidence of individual components of MS, such as BP and fasting glucose.

## Methods

### Study subjects and design

This longitudinal study was performed using a previously described cohort^8^. Briefly, the initial cohort for this study consisted of 5,100 subjects who completed a comprehensive health check-up, including abdominal fat computed tomography (CT) and laboratory exams, from March 2007 to December 2008 at Seoul National University Hospital Healthcare System Gangnam Center. In total, 2,519 subjects who did not complete any voluntary follow-up exams between 2011 and 2013 were excluded from this study. Of the remaining 2,581 subjects, 617 who had MS at baseline were excluded. Finally, a total of 1,964 subjects were enrolled in this study. The median follow-up time for this cohort was 4.5 years. This study’s protocol was approved by the Institutional Review Board of Seoul National University Hospital (IRB No. 0909-012-294) and conformed to the ethical guidelines of the 1975 Declaration of Helsinki. As the researchers accessed only de-identified databases for analytical purposes, the Institutional Review Board waived the need for informed consent.

### Anthropometric and laboratory measurements

The methods applied in this cohort have been described in detail elsewhere^8, 35, 36^. Briefly, each subject underwent an anthropometric assessment and laboratory examination and completed a questionnaire that collected information on the subject’s past medical history; current medication information; and lifestyle, including smoking, alcohol consumption, physical activity, and diet. Height and body weight were measured using Inbody 720® (Biospace, Korea), and BMI was calculated. WC was measured by a well-trained nurse at the midpoint between the lower costal margin and the iliac crest. Blood pressure (BP) was measured twice in a sitting position after at least a 10-min rest. Subjects with a systolic BP ≥140 mmHg or a diastolic BP ≥90 mmHg or the current use of an antihypertensive medication were defined as having hypertension. Fasting plasma glucose levels ≥126 mg/dL or treatment with a hypoglycemic agent or insulin were used to define diabetes mellitus. Current smokers were defined as those who had smoked at least one cigarette per day in the past year. Ex-smokers were defined as subjects who used to smoke cigarettes regularly^37^. Excessive alcohol consumption was defined as >30 g/day for men and >20 g/day for women^38^ Women were considered menopausal if they had not had their periods for over 1 year. Additionally, we used a previously described method to assess physical activity and soft drink consumption^38^. Physical activity was measured using a modified Korean version of the physical activity questionnaire from the National Health and Nutrition Examination Survey, which employs a well-established metabolic equivalent (MET) quantification of physical activity. Briefly, subjects were asked about the type of physical activity that they engaged in, the frequency per week and the duration of each session of physical activity (minutes). We then determined the level of physical activity according to MET-minutes, a well-known parameter. The MET-minutes per week were calculated by multiplying the MET value by the minutes per week spent engaged in physical activity^37^. A diet questionnaire assessed soft drink consumption (≥2/week).

We defined several variables to reflect the changes that occurred during follow-up: new smoking, alcohol intake and soft drink consumption were defined as a new current smoker and a subject with new onset of excessive alcohol consumption and soft drink consumption, respectively, at follow-up.

Blood samples were obtained from an antecubital vein after more than 12 hours of fasting. The serum levels of fasting glucose, serum total cholesterol, serum triglycerides (TG), serum HDL cholesterol, and fasting insulin were measured. All laboratory tests were conducted using standard methods. The follow-up evaluations utilized the same procedures, protocols, and laboratories.

### Measurement of abdominal adipose tissue

Detailed descriptions of the methods used to measure the abdominal adipose tissue area have been published previously^39^. Briefly, a 5-mm thick umbilical level section from a 16-detector row CT scanner (Somatom Sensation 16; Siemens Medical Solutions, Forchheim, Germany) was obtained. The cross-sectional area (cm^2^) of abdominal fat was calculated using Rapidia 2.8 CT software (INFINITT, Seoul, Korea) by setting the attenuation values for a region of interest within a range of −250 to −50 Hounsfield units. The VAT area was defined as intraabdominal fat bound by parietal peritoneum or transversalis fascia, and the SAT area was calculated by subtracting the VAT area from the total adipose tissue area.

### Definition of MS

MS was defined using the modified definition outlined in the National Cholesterol Education Program Adult Treatment Panel III guidelines^40^ following the WC criteria proposed by the WHO’s Regional Office for the Western Pacific Region^41^: (1) WC ≥90 cm in men and 80 cm or more in women; (2) fasting TG ≥150 mg/dL or drug treatment for elevated TG; (3) HDL cholesterol <40 mg/dL in men and less than 50 mg/dL in women or drug treatment for low HDL cholesterol; (4) BP ≥130/85 mmHg or taking antihypertensive medication; and (5) fasting glucose ≥100 mg/dL or taking antidiabetic medication.

### Statistical analysis

The outcome of this study was the development of MS. The baseline characteristics of the participants according to the presence of incident MS were compared using Student’s *t*-test and Pearson’s chi-squared test. Cox proportional hazards models were used to analyze the adjusted HR and 95% CI for incident MS and the individual components of MS per sex-specific 1-SD increase in the VAT and SAT areas and for each sex-specific quintile of difference in the VAT and SAT areas after controlling for potential confounders, which were chosen by statistical significance and clinical importance. The HR per 1-SD was used to compare the relative strength of the relationship across variables in Tables 2, 3 and 4, as the outcome number of the analysis of subjects without any individual MS components at baseline (Table 3) was relatively low in certain quintiles of the cohort, which might have influenced the results. We included change in WC to reflect abdominal adipose tissue changes and also incorporated other lifestyle changes in the final model. Statistical analyses were conducted using SPSS 18.0 (SPSS, Inc., Chicago, IL, USA) and STATA 13.0 (STATA Corp, College Station, TX, USA) software. Statistical significance was defined by a two-tailed p-value of <0.05.

## Electronic supplementary material

supplementary tables
