# Supplementary material for: Body Fat Distribution and the Risk of Incident Metabolic Syndrome: A Longitudinal Cohort Study
Source: Sci Rep. 2017 Sep 8;7:10955. doi: 10.1038/s41598-017-09723-y (PMC5591218; doi:10.1038/s41598-017-09723-y)
Supplement: Supplementary file 1 — supplementary tables [file 41598_2017_9723_MOESM1_ESM.pdf]

Body Fat Distribution and the Risk of Incident Metabolic Syndrome: A Longitudinal Cohort Study

Hyuktae Kwon, M.D., Ph.D. [1,2], Donghee Kim, M.D., Ph.D. [3,4], Joo Sung Kim, M.D., Ph.D. [3]

[1]*Department of Family Medicine, Healthcare Research Institute, Seoul National University Hospital Healthcare System Gangnam Center, Seoul, Korea*

[2]*Department of Family Medicine, Seoul National University Hospital & College of Medicine, Seoul, Korea*

[3]*Department of Internal Medicine, Healthcare Research Institute, Seoul National University Hospital Healthcare System Gangnam Center, Seoul, Korea*

[4]*Division of Gastroenterology and Hepatology, Stanford University School of Medicine, Stanford, California, United States*

Running title: Body Fat Distribution and Incident Metabolic Syndrome

Corresponding Author: Donghee Kim, M.D., Ph.D.

Department of Internal Medicine, Healthcare Research Institute, Seoul National University Hospital Healthcare System Gangnam Center, 39th FL., Gangnam Finance Center, Yeoksam-Dong, Gangnam-Gu, Seoul, 135-984, South Korea

Supplementary Table 1. Main baseline characteristics of the study population and comparison between available and unavailable subjects (n=5,100)

	Follow up data available (n=2,581)	Follow up data absent (n=2,519)	*P*-value
Age (years)	51.1±8.9	51.1±10.3	0.899
Male (%)	1731 (53.1)	1528 (46.9)	<0.001
Smoking (%)	873 (33.8)	693 (27.5)	0.125
Diabetes mellitus (%)	169 (6.5)	216 (8.6)	0.006
Hypertension (%)	534 (20.7)	529 (21.0)	0.785
BMI (kg/m2)	23.94±2.87	24.00 ± 3.12	0.463
WC (cm)	85.78±7.89	85.81 ± 8.39	0.883
Cholesterol (mg/dL)	192.7±33.1	193.4 ± 34.9	0.472
TG (mg/dL)	118.1±71.2	121.3 ± 82.4	0.129
Fasting glucose (mg/dL)	96.8 ± 18.5	97.4 ± 21.9	0.239
HOMA index	2.19 ± 1.23	2.18 ± 1.26	0.672
TAT (cm2)	268.6 ± 90.5	272.6 ± 99.5	0.131
VAT (cm2)	119.5 ± 54.4	116.5 ± 56.1	0.052
SAT (cm2)	149.1 ± 57.7	156.1 ± 65.1	<0.001
MS (%)	617 (23.9)	696 (27.6)	0.002

The data are shown as the mean±SD or n (%).   .

Supplementary Table 2. Multivariable analyses of the risk for incident MS in subjects without MS at Baseline (n=1,964)

	Multivariable model 3		Multivariable model 4	
	HR (95% CI)	*P*-value	HR (95% CI)	*P*-value
VAT				
1st	1	<0.001*		0.002*
2nd	1.47 (0.92-2.37)	0.110	1.77 (1.17-2.67)	0.006
3rd	1.82 (1.13-2.93)	0.013	2.10 (1.41-3.13)	<0.001
4th	2.19 (1.36-3.53)	0.001	2.52 (1.69-3.77)	<0.001
5th	2.54 (1.51-4.28)	<0.001	3.04 (2.00-4.62)	<0.001
VAT (per 1 SD)	1.38 (1.18-1.61)	<0.001	1.50 (1.29-1.74)	<0.001
SAT				
1st	1	0.167*		0.802*
2nd	0.88 (0.58-1.34)	0.555	0.90 (0.64-1.28)	0.566
3rd	1.05 (0.70-1.59)	0.808	1.10 (0.72-1.39)	0.994
4th	1.04 (0.68-1.61)	0.846	1.15 (0.83-1.59)	0.401
5th	1.33 (0.81-2.18)	0.261	1.13 (0.79-1.62)	0.495
SAT (per 1 SD)	1.04 (0.87-1.24)	0.659	0.96 (0.81-1.14)	0.641

**P*-value for test of trend of odds

The multivariable model 3 included baseline fasting triglyceride, high-density lipoprotein cholesterol, fasting glucose, systolic blood pressure, and change in waist circumference in addition to the variables addressed in model 1 of main table 2

The multivariable model 4 included change in waist circumference and HOMA index in addition to the variables addressed in model 1 of main table 2
